# A grid-enabled web service for low-resolution crystal structure refinement

**DOI:** 10.1107/S0907444912001163

**Published:** 2012-02-14

**Authors:** Daniel J. O’Donovan, Ian Stokes-Rees, Yunsun Nam, Stephen C. Blacklow, Gunnar F. Schröder, Axel T. Brunger, Piotr Sliz

**Affiliations:** a Department of Biological Chemistry and Molecular Pharmacology, Harvard Medical School, Boston, MA 02115, USA; b Department of Cancer Biology, Dana Farber Cancer Institute, Boston, MA 02215, USA; c Department of Pathology, Brigham and Women’s Hospital, Boston, MA 02115, USA; d Forschungszentrum Jülich, Institute of Complex Systems (ICS-6), 52425 Jülich, Germany; e Howard Hughes Medical Institute and Departments of Molecular and Cellular Physiology, Neurology and Neurological Sciences, Structural Biology and Photon Science, Stanford School of Medicine, J. H. Clark Center E300C, 318 Campus Drive, Stanford, CA 94305, USA; f Laboratory of Molecular Medicine, Children’s Hospital, Boston, MA 02115, USA

**Keywords:** deformable elastic network restraints, low-resolution refinement, DEN refinement

## Abstract

Deformable elastic network (DEN) restraints have proved to be a powerful tool for refining structures from low-resolution X-ray crystallographic data sets. Unfortunately, optimal refinement using DEN restraints requires extensive calculations and is often hindered by a lack of access to sufficient computational resources. The DEN web service presented here intends to provide structural biologists with access to resources for running computationally intensive DEN refinements in parallel on the Open Science Grid, the US cyberinfrastructure. Access to the grid is provided through a simple and intuitive web interface integrated into the SBGrid Science Portal. Using this portal, refinements combined with full parameter optimization that would take many thousands of hours on standard computational resources can now be completed in several hours. An example of the successful application of DEN restraints to the human Notch1 transcriptional complex using the grid resource, and summaries of all submitted refinements, are presented as justification.

## Abstract 2

The deformable elastic network (DEN) method for reciprocal-space crystallographic refinement improves crystal structures, especially at resolutions lower than 3.5 Å. The DEN web service presented here intends to provide structural biologists with access to resources for running computationally intensive DEN refinements.

## 1. Introduction

**Figure 1:**
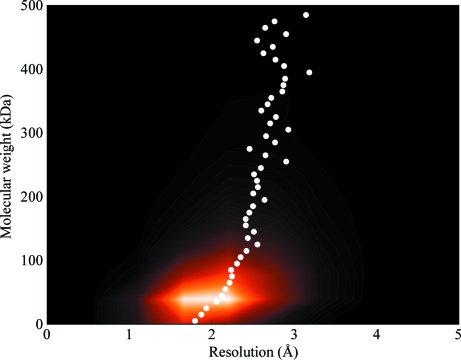
Heat map showing how (X-ray-determined) Protein Data Bank (PDB) entries trend with data resolution and molecular weight. There are many structures recorded between 1.5 and 2.5 Å resolution with a molecular weight of around 40 kDa, indicated by a lighter color. The white dots represent the mean average resolutions for structures in 10 kDa ranges. This figure shows that larger molecules tend to have poorer resolutions. As the molecular weight of structures increases, the mean resolution of the recorded X-ray data also increases. The PDB only contains structures that have been finalized; therefore, there may be many more low-resolution data sets from crystals that did not yield publication-quality structures.

Larger macromolecules tend to produce crystals that diffract weakly (Fig. 1 ▶). As interest in these large assemblies grows, the range of methods for determining structures from these weakly diffracting crystals is also growing. Refinement of structures at resolutions lower than 3.5 Å is known to be difficult and error-prone, although careful investigations can produce good results (Brunger *et al.*, 2009 ▶). Often, it is more convenient for structural biologists to pursue a ‘divide-and-conquer’ approach in which fragments of a larger molecule are solved independently and then used to reconstruct the larger assembly. Using a threshold of 3.5 Å resolution, there are approximately 1000 structures in the Protein Data Bank (Berman *et al.*, 2000 ▶) which fall into this category. These structures represent less than 2% of the 68 140 total structures currently in the database. It is believed that the advent of new computational methods for low-resolution refinement will allow more structures to be refined from data recorded at resolutions that were previously believed to be unusable.

The deformable elastic network (DEN) method for reciprocal-space crystallographic refinement improves crystal structures, especially at resolutions lower than 3.5 Å and in cases where the initial structure is far from the target (Schröder *et al.*, 2010 ▶). In this resolution range, overfitting becomes a significant problem because the number of experimental observations (Bragg intensities) can be smaller than the number of free torsion angles (*e.g.* for a protein, ϕ and ψ backbone torsion angles and rotatable side-chain torsion angles) that the refinement process attempts to fit. The DEN approach reduces overfitting by using a reference-model structure. This reference model is typically a homology model or a structure of the protein in a different conformation, or simply the starting structure for the refinement. A set of harmonic distance restraints are defined for a sparse set of randomly chosen pairs of atoms in the reference model, creating an elastic network. During the refinement process the equilibrium distances of the harmonic restraints are allowed to deform so as to better fit the diffraction data.

The DEN-refinement process is controlled by several important parameters that can affect the success of the method. Those parameters include a weight (ω_DEN_) that determines the effect of the DEN restraints on the refinement relative to the other terms of the target function used for refinement, the deformation factor γ_DEN_ and the simulated-annealing temperature. The ω_DEN_ parameter can range from 0 to 300. When ω_DEN_ = 0 the DEN restraints have no effect on the refinement process, and when ω_DEN_ = 300 and γ_DEN_ = 0 the DEN term is high enough to effectively restrain the refined structure to the reference model. A suitable ω_DEN_ in the range between 0 and 300 will need to be determined in each case and will depend on the overall similarity between the refined structure and its reference model. The γ_DEN_ parameter can range from 0 to 1 and at larger γ_DEN_ values the DEN distances can eventually significantly deviate from the reference model during the DEN-refinement process. This deformation is justified through the observation that local protein structure tends to evolve at a slower rate than sequence and function (Schröder *et al.*, 2010 ▶).

In addition, for each parameter combination several refinement repeats should be performed with different initial random-number seeds for the simulated-annealing process and the random selection of DEN distances, since DEN refinement is an inherently stochastic search process. Currently, optimal choices for these values should be obtained by an exhaustive grid search of the parameter space, although in the future guidelines may be developed to make the search more efficient based on growing experience with the DEN method. In a complete parameter search many thousands of individual parameter choices must be evaluated and each run must be executed to completion. Depending on the number of parameters being searched and the range and accuracy desired in these parameters, DEN refinements often result in many thousands of independent refinement calculations. A single refinement calculation (with one set of parameters) may require several hours of serial processing time on a modern processor depending on the unit-cell content and the limiting resolution of the crystal, with the implication that a full DEN computation could require of the order of one year of serial computational time for the largest macromolecular crystal structures. Such computational demands put DEN beyond the capacity of desktop computing resources and require access to large computer clusters.

Grid computing provides an attractive approach to support the widespread implementation of DEN refinement. We have previously developed the SBGrid Science Portal (http://sbgrid.org) to provide a standard framework that can support specialized structure-determination computations such as wide-search molecular replacement (Stokes-Rees & Sliz, 2010 ▶). The computational power of the portal is provided by the extensive opportunistic resources of the Open Science Grid (OSG; Pordes *et al.*, 2007 ▶). OSG federated resources include large computing clusters at over 50 research institutions; major computational sites utilized by SBGrid include the University of Nebraska-Lincoln, Purdue University and Clemson University. The science portal consists of a Django-driven web interface with LDAP user authentication allowing SSH, WebDAV and S/FTP access to data. The grid interface is powered by Condor (Thain *et al.*, 2005 ▶) and is linked to the portal through the *django-condor* application. The Virtual Data Toolkit (Roy, 2009 ▶) and GlideinWMS (Sfiligoi *et al.*, 2009 ▶) through the GlideIn factory at the University of California at San Diego allow many thousands of unique Condor tasks to be processed through the portal every day.

In this paper, we report an extension of the SBGrid Science Portal to support the DEN-refinement method. The DEN web service can complete an entire DEN refinement with full parameter search within ∼10 h (when grid resources are not saturated) through the utilization of OSG resources. A web interface has been developed that allows users to upload the required data files [a user-edited *CNS* (Brünger *et al.*, 1998 ▶) refine_den.inp refinement script and required diffraction data and models] and control other parameters that determine the extent of the grid search. Upon completion of all refinements, the results are presented to the user in a sortable table through the web interface with a series of heat-map plots. The DEN web service was initially tested for its utility in refinement of a Notch transcriptional complex at 3.85 Å resolution and has been further tested and validated by DEN refinements submitted by users. A blind analysis of these initial runs was performed and indicated a significant improvement in *R*_free_ values for most submitted jobs. The SBGrid Science Portal will make the method of DEN refinement immediately available to the structural biology community and will also provide a wealth of information useful for further optimization of the DEN method.

## 2. Submitting DEN-refinement jobs

### 2.1. Registration

The SBGrid Science Portal requires that all users register. Registration is needed to ensure legitimate and secure use of global computing resources and allows users to access a secure workspace for all their portal-based data and com­putations. Owing to the length of time required for DEN refinements, the account system allows users to return to the portal and track the progress of one or more previously submitted computations.

### 2.2. Preparation

A DEN-refinement optimization requires a diffraction data set, a coordinate file with an initial model and a coordinate file with a reference structure (which could also be the initial model). The initial model is typically obtained by molecular replacement or by the fitting of electron-density maps phased by experimental phasing methods and can combine molecular-replacement fragments and *de novo* modeled structures. The reference structure can be provided by a previously refined higher resolution coordinate file with an identical or a related sequence or a model generated by software such as *Desmond* (Bowers *et al.*, 2006 ▶) or *Modeller* (Eswar *et al.*, 2006 ▶). In general, the reference structure represents the prior knowledge of the structure. Reference structures can be in an arbitrary orientation and position since DEN distances are invariant with respect to the placement of the reference model. If the reference model consists of several domains or independent molecules, the relative placement of these components can be arbitrary as well if the default-sequence and chain-separation selections are used (Schröder *et al.*, 2010 ▶). However, if the distance selections are sparse random drawings from all possible interatomic distances then the relative placements of the components in the reference model are important.

Two mandatory files need to be prepared by the user before portal submission: a DEN-refinement input file based on the template refine_den.inp from the DEN tutorial of the *CNS* website and included with the *CNS* v.1.3 distribution, and an archive file containing all the data files referenced by the *CNS* input file, including the diffraction data set, the initial model and reference coordinates and any other files that are required by the refinement job, such as special parameter files or noncrystallographic symmetry files. The DEN-input file must be modified to reference the diffraction data set, the initial model and the reference structure. All values in this input file are conserved during the DEN optimization as are parameters such as the starting temperature for the simulated annealing (*temperature*) or the number of refinement macrocycles. The archive file can be in any common file-compression format. Detailed instructions on how to prepare the DEN-input file can be found in the DEN tutorial on the *CNS* website (http://www.cns-online.org). Once the DEN-input file and all accessory files have been prepared, running a single iteration of DEN refinement on a local machine is highly recommended in order to ensure that the file is correct and does not produce errors.

### 2.3. Submission and monitoring

**Figure 2:**
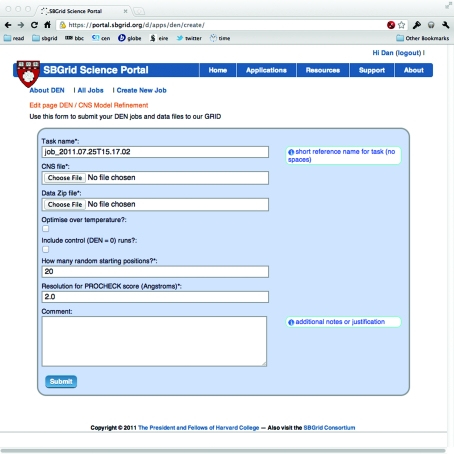
DEN Portal Application job-submission interface. This is the form through which registered users can submit new tasks to the Open Science Grid. The form requires a unique ‘task name’, DEN input file, data archive and the resolution of the diffraction data for *PROCHECK* validity checking. There are other optional parameters.

The DEN-refinement computation is initiated through the SBGrid Science Portal, which provides a web form to name and describe the computation, upload necessary files prepared as described in §2.2 and provide other computation configurations (see Fig. 2 ▶). The uploaded files are checked for correctness and consistency.

The web form also provides an option to perform an additional annealing-temperature optimization (the default is to use the temperature specified in the DEN-refinement input file). There are also input fields for the number of initial random-number seeds (for the velocity assignments of the simulated-annealing runs and the random selection of DEN distances) and for setting the resolution of the diffraction data for the *PROCHECK* (Laskowski *et al.*, 1993 ▶) score.

Submitting the form uploads the data and parameters, performs the checks and, provided the checks pass, queues the computation for execution on OSG. Grid execution is monitored *via* the *django*–*condor* application bridge and when all jobs have finished executing, results are generated and the user is notified by e-mail.

### 2.4. DEN optimization

DEN-refinement parameter optimization follows the originally described brute-force strategy (Schröder *et al.*, 2010 ▶). This takes all permutations of each parameter over a fixed range of values, conventionally known as a ‘parameter sweep’. The optimized parameter set is that which will lead to the lowest *R*_free_ (Brünger, 1992 ▶) value after the DEN refinement (among the multiple repeat refinements for each particular set, the refinement with the lowest *R*_free_ will be considered for that particular set). In addition, a *PROCHECK* validation is performed to provide extra assurance of the validity of the refined structure through the Ramachandran percentage ‘disallowed’ score and to enable the user to choose between structures that have similar *R*_free_ values.

The parameters being varied to produce an optimal *R*_free_ in a DEN run are γ_DEN_, ω_DEN_ and *n*_seed_, the number of unique seed values for the random-number generator. The special case of ω_DEN_ = 0 is known as ‘no DEN’ and can be used as a control for comparing results when the DEN restraints are turned off. Optionally, temperature can also be varied to optimize *R*_free_; doing this will perform DEN refinements for a range of starting temperatures.

Following Schröder *et al.* (2010 ▶), the DEN-refinement optimization protocol uses (0, 0.25, 0.5, 0.75, 1.0) for the γ_DEN_ parameter and six values of ω_DEN_ (3, 15, 30, 60, 150, 300). As the DEN-refinement process is inherently stochastic, it is important to test a range of randomly chosen random-number seeds for the refinement, which has been set to a default value of 20 seeds. This search space for parameter optimization for DEN refinement covers 600 individual refinement computations (5 × 6 × 20). When the *temperature* parameter is turned on, runs are started at one of six different starting temperatures (300, 600, 1200, 2000, 2500, 3000), requiring 3600 refinements. If the control (DEN = 0) is included, an additional *n*_seed_ refinements are performed with ω_DEN_ = 0 and γ_DEN_ = 0 and the default temperature in the input file.

### 2.5. Analyzing output and finishing refinement

Once the DEN-refinement task has completed, the user is sent a notification e-mail containing a link to the results page. The results will consist of a set of heat maps showing the best *R*_free_ under different parameter conditions, a table displaying all salient results and output data files. For each given *temperature*, γ_DEN_ and ω_DEN_ there will be *n*_seed_ repeated jobs; the heat map presents the best *R*_free_ chosen from these *n*_seed_ runs (blue implies a lower *R*_free_ and red a higher *R*_free_). The Ramachandran heat map presents the percentage of residues in the disallowed region of the Ramachandran plot (from the structure with the best *R*_free_ value for each γ_DEN_, ω_DEN_ parameter pair). The results from all runs are presented in a sortable table, which also includes html links to the refined coordinates, Fourier coefficients for map calculations and *PROCHECK* summary files. To save load times, initially the best 50 results (in terms of *R*_free_) are presented, but a link to the entire results list is provided. From these collected results, the user is able to choose the most promising (typically based on lowest *R*_free_) and use this for subsequent remodeling and refinement cycles. Additional rounds of DEN refinement can be performed as needed, although most of the benefit of DEN refinement is generally observed in the first round when the initial model is still far from the target structure.

## 3. Example: Notch transcriptional complex

**Figure 3:**
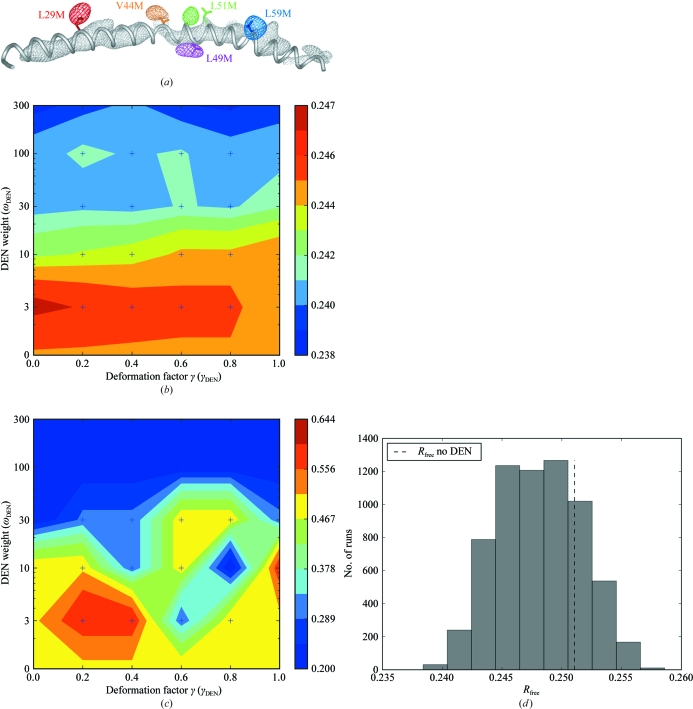
Test case: a Notch1 transcription complex containing the RAM region. (*a*) The first 4.2–7 Å low-resolution structure of a human Notch1 complex consisting of the Notch ankyrin-repeat domain, the CSL transcription factor and the Mastermind-like 1 (MAML-1) co-activator was determined by combining molecular replacement and selenomethionine scanning. Single Leu-to-Met or Val-to-Met mutations (labeled with the MAML-1 residue number) were introduced into the MAML-1 polypeptide for incorporation of selenomethionine. Anomalous Fourier difference maps were calculated for each of five mutants (the high-resolution limit for each data set was between 6 and 7.5 Å) using the anomalous signal from selenomethionine and the phase calculated by molecular replacement. Each map shows a clear peak at the predicted location of the mutated residue, indicated by the matching colors. The gray mesh represents the density for the MAML-1 as part of a 2*F*_o_ − *F*_c_ density map calculated without any atoms modeled for the MAML-1 helix. Adapted from the supplementary information in Nam *et al.* (2006 ▶). (*b*) An *R*_free_ heat map of results from the Notch protein DEN optimization using an initial starting temperature of 1000 K. The minimal *R*_free_ values for each parameter pair (ω_DEN_ and γ_DEN_) over multiple refinement repeats are shown. (*c*) The corresponding Ramachandran statistics (percentage of disallowed backbone angles). For each parameter pair, the structure with the lowest *R*_free_ value was usen to calculate the Ramachandran statistics. (*d*) A histogram showing all calculated *R*_free_ for a complete portal DEN optimization of the Notch1 complex. The dashed line is the best that could be achieved without DEN (*i.e.*, the lowest *R*_free_ of the all the ‘no DEN’ repeat refinements); the lowest (or best) *R*_free_ chosen is the far left tail of the histogram.

Activated Notch signaling is important for various cell-fate decisions and leads to the assembly of a multi-protein complex on DNA that can promote transcription of downstream genes. The structure of a human Notch1 transcriptional complex was initially determined using anisotropic diffraction at a resolution between 4.2 and 7 Å by combining molecular replacement with selenomethionine-scanning mutagenesis (Fig. 3 ▶ *a*). Ultimately, crystals diffracting to 3.25 Å resolution were obtained and the refined coordinates confirmed the essential architectural features of the earlier 4.2–7 Å model (Nam *et al.*, 2006 ▶). More recently, we crystallized a Notch transcriptional complex in the presence of the RAM region of Notch1, which engages the transcription factor (called CSL) at a different site from the ankyrin-repeat domain of Notch1 present in the previously reported structure (Wilson & Kovall, 2006 ▶; Friedmann *et al.*, 2008 ▶). The 3.85 Å resolution data set produced from these crystals was used to test the portal-based DEN-refinement optimization protocol. Over 28 000 h of com­puting time was used to complete this DEN run in 8 h, a 3500-fold speedup over serial computation. The heat maps for the refined coordinates are presented in Figs. 3 ▶(*b*) and 3 ▶(*c*). These figures show that the best refinements occurred with higher values of ω_DEN_ and with a γ_DEN_ of 0.8. The high values of ω_DEN_ imply that the reference model provided a good template for the RAM structure, although flexibility was still required to allow adjustments. Indeed, the C^α^ root-mean-square deviation between the refined coordinates and the reference model is only 0.38 Å and differences between the two structures are limited to loop regions. A detailed description of the complex structure and relevant biochemistry is presented separately (Choi *et al.*, 2012 ▶). A histogram showing all of the *R*_free_ scores calculated from a complete DEN optimization of the Notch1 complex is shown in Fig. 3 ▶(*d*).

## 4. Current status

**Figure 4:**
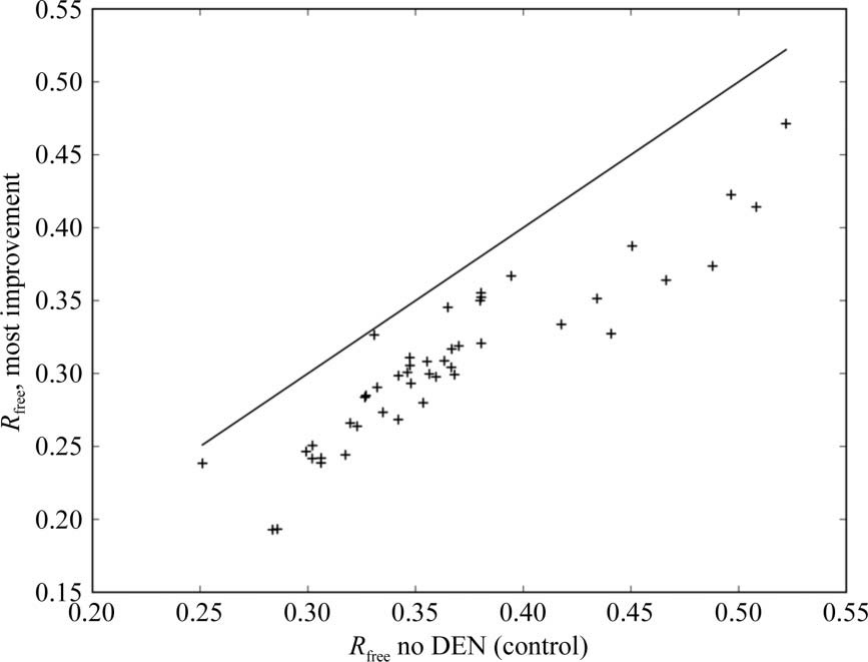
Chart showing *R*_free_ (optimum over all results) and *R*_free_ (no DEN; optimum over all control refinements without DEN) for all tasks submitted by users. Each point lies below the diagonal line, showing that all results demonstrated an improvement in *R*_free_ over the control results.

To date, more than 60 unique DEN-refinement optimizations with different structures or refinement scripts have been submitted through the SBGrid Science Portal by ten researchers. More than 80 years of CPU execution time have been used by these jobs. Most values in the portal DEN refinement resulted in an improvement in *R*_free_, but a significant number of values showed no improvement in *R*_free_. Fig. 4 ▶ is a scatter chart showing each task submitted and completed on the DEN portal to date. The *x* axis corresponds to the resulting *R*_free_ scores when run with ω_DEN_ = 0 and γ_DEN_ = 0, *i.e.* control or ‘no DEN’ runs; the *y* axis shows the most improved values of *R*_free_. The diagonal line plots where *R*_free_ (optimum) is equal to *R*_free_ (no DEN). As all points lie below this line, we see that all tasks showed an improvement in *R*_free_.

## 5. Implementation

The public-serving frontend to the SBGrid Portal is written in Python using the Django web framework. The DEN code base consists of a Python-written Django application (or ‘app’) that integrates with the existing Portal code and negotiates with the Condor job scheduler *via* the *django–condor* application, also written in Python. Through an Apache process running on the SBGrid Portal, the Django-based website allows the DEN application to serve the DEN web pages. Python code integrated into the DEN application verifies and checks the input data files and user input data, creates submission scripts, launches DEN jobs on the grid and collects and processes the resultant information from DEN jobs. The application also updates a MySQL database (MySQL AB, Uppsala, Sweden; http://www.mysql.com) which is used for logging and tracking all tasks. The code was written using the Enthought Python 2.7 distribution and Django 1.3.1, although earlier versions (to v.2.5) of Python are expected to function correctly.

The code is available for inspection from the SBGrid Portal home page (http://portal.sbgrid.org) and relies on portions of the Portal code base.

## 6. Conclusions

Many multicomponent biologically important complexes can be crystallized, but structural interpretation is often limited by low-resolution diffraction data. The DEN-refinement optimization provides a useful tool for interpretation of diffraction data that might otherwise be uninformative. The optimization protocol provides refined models with optimal *R*_free_ values and geometry-validation scores that guide further refinement strategies. In the majority of completed DEN tasks, including the initial refinement of the Notch transcriptional complex, the grid DEN refinement resulted in a further decrease in *R*_free_ compared with the starting structure and compared with controls without DEN restraints.

The DEN portal provides an interface with the vast computing resources available on the grid. There are several barriers that users must hurdle before being able to execute a successful DEN refinement. DEN refinements rely on a suitable model with prior structural information. When there is no known structure of high similarity a model must be generated, the production of which can be difficult and error-prone. To completely master the refinement steps, a familiarity with the *CNS* program is needed; the DEN portal only performs *CNS* refinement tasks. Thus, the DEN refinement must be tested before submitting it to the DEN portal.

An additional consequence of calculating structures using DEN restraints is likely to emerge from consideration of the potentially interesting patterns found in the DEN refinements submitted by the user community. Through investigation of these results and patterns, more will eventually be understood about the optimal conditions for DEN refinement, leading to a less computationally intensive method for optimizing these parameters in the future. For example, it seems that more than half of the search space rarely produces good results, and similar results are often obtained for fairly broad regions. By performing line searches rather than full two- or three-dimensional searches, the overall computational cost of the experiment could be significantly reduced while still obtaining a close-to-optimum solution. Thus, it may be possible to reduce the DEN-parameter optimization to something that can run on a desktop computer within a reasonable time frame.

Even the smallest parameter searches suggested by Schröder *et al.* (2010 ▶) demand thousands of DEN refinements. Researchers will often increase this number in the search for an optimal structure. The resulting computational workflows are simply impossible to calculate using a single modern personal computer. It is believed that the grid technology presented in this paper will be an important tool for researchers to determine good-quality structures from low-resolution diffraction data. Data sets that have previously been discarded because there was no method for producing reasonable structures can now be revisited using the DEN portal.
